# Activity of aztreonam–avibactam and comparators against difficult-to-treat resistant Enterobacterales from United States medical centres (2020–24) ^†^

**DOI:** 10.1093/jacamr/dlag249

**Published:** 2026-11-05

**Authors:** Helio S Sader, John H Kimbrough, Dmitri Debabov, Krisztina M Papp-Wallace, Mariana Castanheira

**Affiliations:** Element Iowa City (JMI Laboratories), 345 Beaver Kreek Centre, Suite A, North Liberty, IA 52317, USA; Element Iowa City (JMI Laboratories), 345 Beaver Kreek Centre, Suite A, North Liberty, IA 52317, USA; AbbVie Inc., Chicago, IL, USA; Element Iowa City (JMI Laboratories), 345 Beaver Kreek Centre, Suite A, North Liberty, IA 52317, USA; Element Iowa City (JMI Laboratories), 345 Beaver Kreek Centre, Suite A, North Liberty, IA 52317, USA

## Abstract

**Background:**

The occurrence of infections caused by Gram-negative bacteria with difficult-to-treat resistance (DTR) is rapidly increasing, generally requiring the use of recently approved, last-resort antimicrobial agents or old compounds that are more toxic and/or less effective. We evaluated the activity of aztreonam–avibactam and comparators against DTR Enterobacterales.

**Methods:**

A total of 42 295 Enterobacterales isolates were consecutively collected (one per patient) from 85 US medical centres in 2020–24 and susceptibility tested by Clinical Laboratory Standards Institute broth microdilution. The collection included 450 carbapenem-resistant Enterobacterales (CRE) and 284 DTR isolates, which were screened for β-lactamases by whole genome sequencing.

**Results:**

Aztreonam–avibactam was active against 98.2% of DTR (minimum inhibitory concentration for 50%/90% of the collection, 0.25/1 mg/L) and 97.1% of CRE isolates and retained potent activity (≥90.0% susceptibility) against DTR isolates non-susceptible to ceftazidime–avibactam, meropenem–vaborbactam and/or cefiderocol. Cefiderocol was active against 92.8% of DTR isolates, whereas ceftazidime–avibactam, meropenem–vaborbactam, imipenem–relebactam and the aminoglycosides exhibited limited activity against these organisms. Aztreonam–avibactam and cefiderocol were active against 97.8% and 84.4% of DTR metallo-β-lactamase (MBL) producers, respectively. The most common carbapenemase genes identified among DTR isolates were *bla*_KPC_ (57.7% of isolates) and *bla*_NDM_ (29.9%). An MBL gene was observed in 31.7% of DTR and 25.1% of CRE isolates.

**Conclusions:**

Aztreonam–avibactam retained potent activity against DTR Enterobacterales from US medical centres, and its activity was not adversely affected by clinically relevant carbapenemases or resistance to agents used to treat multidrug-resistant Enterobacterales. The activities of other β-lactam/β-lactamase inhibitor combinations and cefiderocol were compromised by the increased occurrence of MBL producers.

## Introduction

Difficult-to-treat resistance (DTR) in Gram-negative bacteria is defined as non-susceptibility to all first-line agents, including all β-lactams and fluoroquinolones.^1,2^ First-line agents represent those antibiotics that are more effective and less toxic; thus, non-susceptibility to all first-line agents leads to both inappropriate empirical therapy and a subsequent need for less effective and/or more toxic agents, such as aminoglycosides, polymyxins and tigecycline. The aminoglycosides and the polymyxins (colistin and polymyxin B) are nephrotoxic and poorly penetrate relevant infection sites.^3^ Tigecycline blood levels are generally lower than the minimal inhibitory concentrations exhibited by most Enterobacterales species. Moreover, tigecycline use has been associated with increased mortality rates and should not be used to treat serious infections when other effective agents are available.^4^

The impact of DTR on patient mortality has been extensively evaluated by the Antimicrobial Resistance Outcomes Research Initiative (ARORI) of the National Institute of Health (NIH). NIH-ARORI assessed 29 474 patients with Gram-negative bloodstream infections (BSI) from 173 US hospitals and found a higher unadjusted mortality rate in patients with DTR (43%) when compared with patients with carbapenem-resistant Enterobacterales (CRE; 35%) isolates, patients with organisms resistant to broad-spectrum cephalosporins (22%) or patients with fluoroquinolone-resistant organisms (18%). Moreover, when compared with BSI caused by non-resistant Gram-negatives, all resistant phenotypes remained independently associated with an increased mortality risk after adjustment for confounders.^1^

A more recent study from NIH-ARORI^2^ evaluated 5056 cases of DTR infections from 262 US hospitals between 2016 and 2023. The authors observed that the use of inappropriate antimicrobial agents (*in vitro* discordant) and the resulting mortality rate were unacceptably elevated among patients with infections caused by DTR Gram-negative bacteria despite the availability of newer antimicrobial agents. Moreover, the authors of the study highlighted the importance of the rapid recognition of DTR organisms as a critical factor in reducing mortality.^2^

The newer β-lactam/β-lactamase inhibitor combinations (BLICs) ceftazidime–avibactam, meropenem–vaborbactam and imipenem–relebactam as well as the new siderophore cephalosporin cefiderocol are stable to hydrolysis by *Klebsiellla pneumoniae* carbapenemase (KPC) and other clinically relevant serine carbapenemases and have been recommended for the treatment of CRE and DTR Enterobacterales in the USA.^5^ However, more recent surveillance data has shown an increase in metallo-β-lactamase (MBL)-producing CRE in some US medical centres. These newer antimicrobial agents, especially the new BLICs listed above, have very limited activity against MBL-producing CRE.^6^ Thus, constant surveillance with large comprehensive programs is essential to understand the current epidemiology and susceptibility profile of CRE and DTR Enterobacterales in US medical centres.^7,8^

Aztreonam–avibactam was recently approved by the US Food and Drug Administration (FDA) for treatment of complicated intra-abdominal infections (cIAI) and infections due to aerobic Gram-negative bacteria in adults with limited treatment options (https://www.accessdata.fda.gov/drugsatfda_docs/label/2025/217906Orig1s000lbl.pdf; accessed 18 May 2026) and by the European Medicines Agency (EMA) in the European Union for treatment of adults with cIAI, complicated urinary tract infection, hospital-acquired pneumonia, including ventilator-associated pneumonia, and infections due to aerobic Gram-negative bacteria in adults with limited treatment options (https://www.ema.europa.eu/en/medicines/human/EPAR/emblaveo; accessed 18 May 2026). This new BLIC has demonstrated potent activity against CRE Enterobacterales worldwide, including MBL producers.^9,10^ In this investigation, we evaluated the activity of aztreonam–avibactam in comparison to ceftazidime–avibactam, meropenem–vaborbactam, imipenem–relebactam and cefiderocol against DTR Enterobacterales from US medical centres.

## Methods

A total of 42 295 Enterobacterales isolates were consecutively collected (one per patient) from 85 US medical centres in 2020–24 via the International Network for Optimal Resistance Monitoring (INFORM) programme.^7^ Medical centres collected a defined number of consecutive isolates (one per infection episode) from designated infection types according to a common study protocol. Only bacterial isolates determined to be significant by local criteria as the reported probable cause of infection were included in the study. Species identification was confirmed by standard biochemical tests and/or using the MALDI Biotyper (Bruker Daltonics, Billerica, MA, USA).

All isolates were susceptibility tested in a monitoring laboratory (Element Iowa City [JMI Laboratories]) by the broth microdilution method as described by the Clinical Laboratory Standards Institute (CLSI).^11,12^ Cefiderocol was tested in iron-depleted media.^11^ The organism collection included 450 CRE and 284 DTR Enterobacterales. CRE was defined as displaying imipenem or meropenem MIC values at ≥4 mg/L and imipenem was not applied to *Proteus mirabilis* or indole-positive Proteeae due to their intrinsically elevated imipenem MIC values. DTR was defined as non-susceptible (intermediate or resistant) to cephalosporins (ceftriaxone, ceftazidime and cefepime), ceftolozane–tazobactam, piperacillin–tazobactam, carbapenems (imipenem and meropenem) and fluoroquinolones (ciprofloxacin and levofloxacin).^1,2,5^ All CRE isolates were screened for β-lactamase–encoding genes by applying genome sequencing and *in silico* screening, as previously described.^13^

## Results

Aztreonam–avibactam was active (MIC ≤4 mg/L) against 98.2% of DTR and 97.1% of CRE isolates (minimum inhibitory concentration for 50%/90% of the collection [MIC_50/90_], 0.25/1 mg/L for both subsets; Table 1) and retained potent activity against DTR isolates non-susceptible to ceftazidime–avibactam (MIC_50/90_, 0.25/1 mg/L; 96.6% susceptible), meropenem–vaborbactam (MIC_50/90_, 0.25/1 mg/L; 97.1% susceptible) and/or cefiderocol (MIC_50/90_, 0.5/4 mg/L; 90.0% susceptible; Table 1). Notably, ceftazidime–avibactam, meropenem–vaborbactam and imipenem–relebactam exhibited limited activity against CRE and DTR isolates (Table 1). Ceftazidime–avibactam was active against 69.4% of DTR and 74.4% of CRE isolates, whereas meropenem–vaborbactam and imipenem–relebactam were active against 63.4% and 60.8% of DTR isolates, respectively, and 70.9% and 66.4% of CRE isolates, respectively (Table 1).

**Table 1.** Antimicrobial susceptibility of selected resistant subsets

Antimicrobial agent	% Susceptible per CLSI or US FDA criteria (no. of isolates)					
	DTR (284)	CAZ-AVI-NS DTR (87)	MEM-VAB-NS DTR (104)	Cefiderocol-NS DTR (20)	MBL-producing DTR (90)	CRE (450)
Aztreonam–avibactam	98.2	96.6	97.1	90.0	97.8	97.1
Ceftazidime–avibactam	69.4	0.0	21.2	20.0	5.6	74.4
Meropenem–vaborbactam	63.4	5.7	0.0	20.0	7.8	70.9
Imipenem–relebactam	60.8	1.1	3.8	15.0	1.1	66.4
Cefiderocol	92.8	81.6	84.6	0.0	84.4	94.0
Gentamicin	53.9	59.8	52.9	65.0	60.0	61.6
Amikacin	55.6	55.2	51.0	65.0	54.4	66.2

DTR, difficult-to-treat resistant; CAZ-AVI, ceftazidime–avibactam; NS, non-susceptible; MEM-VAB, meropenem–vaborbactam; CRE, carbapenem-resistant Enterobacterales.

Cefiderocol was active against 92.8% of DTR and 94.0% of CRE isolates (MIC_50/90_, 1/4 mg/L for both subsets) and retained some activity against DTR isolates non-susceptible to ceftazidime–avibactam (MIC_50/90_, 2/16 mg/L; 81.6% susceptible) and/or meropenem–vaborbactam (MIC_50/90_, 2/16 mg/L; 84.6% susceptible; Table 1). Moreover, cefiderocol was active against 76.9% (10 of 13) aztreonam–avibactam–non-susceptible isolates.

The aminoglycosides gentamicin and amikacin also showed limited activity against these organisms (Table 1). Gentamicin was active against 53.9% of DTR and 61.6% of CRE isolates, and amikacin was active against 55.6% of DTR and 66.2% of CRE isolates. Moreover, these aminoglycosides were active against 51.0%–65.0% of DTR isolates not susceptible to ceftazidime–avibactam, meropenem–vaborbactam and/or cefiderocol (Table 1).

The DTR isolates were from 52 medical centres located in 26 states. States with the highest number of DTR isolates were New York (75 isolates from four medical centres) and Arizona (29 isolates from four centres). Moreover, 84 sequence types were observed among DTR isolates, with the most common being ST258 (*n* = 33 [11.6%]; 18 medical centres from 12 states) and ST24 (*n* = 24 [8.5%]; 12 medical centres from 8 states).

The most common carbapenemase genes identified among DTR (*n* = 284) isolates were *bla*_KPC_ (57.7% of isolates) and *bla*_NDM_ (29.9%). DTR and CRE isolates exhibited similar frequencies of carbapenemase types (Figure 1). *bla*_KPC_ and *bla*_NDM_ were identified in 51.3% and 22.2% of CRE isolates, respectively. An MBL gene was observed in 31.7% of DTR and 25.1% of CRE isolates (Figure 1). The BLICs and cefiderocol were highly active against KPC producers, with susceptibility ranging from 98.2% (imipenem–relebactam) to 100.0% (aztreonam–avibactam). Moreover, aztreonam–avibactam (MIC_50/90_, 0.12/0.5 mg/L) and cefiderocol (MIC_50/90_, 2/8 mg/L) were active against 98.2% and 84.1% of MBL producers, respectively, whereas ceftazidime–avibactam, meropenem–vaborbactam and imipenem–relebactam exhibited limited activity against these organisms (Table 1 and Figure S1, available as Supplementary data at *JAC-AMR* Online). Aztreonam–avibactam, ceftazidime–avibactam and cefiderocol retained good activity (95.5% susceptibility for all three compounds) against the group of isolates that produced carbapenemases other than KPC or MBLs, which included OXA-48-like (14 isolates), SME type (7 isolates) and IMI-4 (1 isolate), and excluded MBL co-producer isolates. Susceptibility of non-carbapenemase–producing CRE varied from 83.7% (imipenem–relebactam) to 93.5% (ceftazidime–avibactam and cefiderocol) and aztreonam–avibactam was active against 89.1% of isolates (Figure S1).

**Figure 1.**
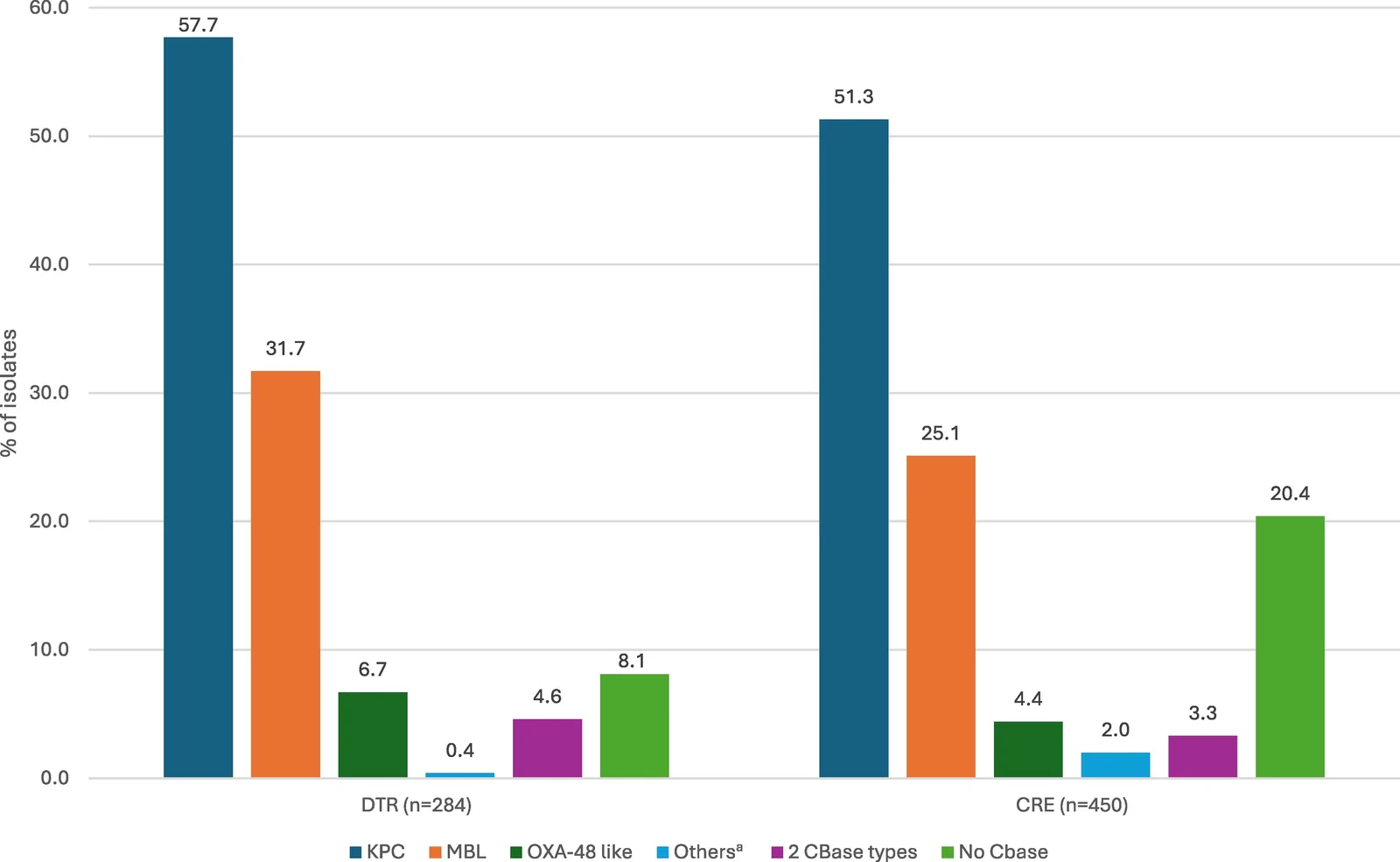
Distribution of carbapenemase types among DTR and CRE isolates. ^a^Includes GES-6 (one isolate) for DTR and SME-2 (five), SME-4 (two), GES-6 (one) and IMI-4 (one) for CRE. DTR, difficult-to-treat resistant; CRE, carbapenem-resistant Enterobacterales; KPC, *Klebsiellla pneumoniae* carbapenemase; MBL, metallo-β-lactamases; OXA, oxacillinase; CBase, carbapenemase.

## Discussion

DTR organisms, which are non-susceptible to all first-line agents, present a treatment challenge due to limited high-efficacy, low-toxicity treatment options and the need to use recently approved last-resort antimicrobial agents or older compounds that are more toxic and/or less effective. Most importantly, the occurrence of infections caused by DTR Gram-negative bacteria is rapidly increasing in US hospitals, and infections caused by these organisms clearly are correlated to increased mortality.^1,2,8^ Tsai *et al*. evaluated the prevalence of Gram-negative bacilli with a DTR phenotype from 10 hospitals in the southeastern US between 2015 and 2020 and reported a significant increase in the prevalence of DTR Enterobacterales from 0.11% to 0.28% (*P* = 0.023).^8^

In this investigation, we evaluated a large collection of Enterobacterales (>40 000 isolates) from 85 US medical centres over 5 years. Our results showed that only the recently approved compounds aztreonam–avibactam and cefiderocol exhibited reasonable activity (>90% susceptibility per CLSI/US FDA breakpoint criteria) against DTR Enterobacterales. Notably, aztreonam–avibactam (MIC_50/90_, 0.25/1 mg/L; 98.0% susceptible) showed higher susceptibility and greater potency (4-fold lower MIC_50/90_ values) than cefiderocol (MIC_50/90_, 1/4 mg/L; 93.5% susceptible per US FDA and 82.4% susceptible per EUCAST.^14^ The BLICs recommended by the Infectious Diseases Society of America guidance (ceftazidime–avibactam, meropenem–vaborbactam and imipenem–relebactam) were active against only 63.2%–71.0% of DTR isolates, and the aminoglycosides were active against only 54.4%–57.0% of these organisms. Another valuable finding of this study was the fact that aztreonam–avibactam maintained activity against isolates not susceptible to ceftazidime–avibactam, meropenem–vaborbactam and/or cefiderocol. In conclusion, our results highlight the continuous changes in the antimicrobial resistance profile and epidemiology of Enterobacterales causing infections in US hospitals and emphasize the importance of continued surveillance through comprehensive programmes.

## Supplementary Material

dlag249_Supplementary_Data

## Transparency declarations

Element Iowa City (JMI Laboratories) was contracted to perform services in 2025 for AbbVie, Inc., AdjuTec Pharma, Affinity Biosensors, AimMax Therapeutics, Amazon.com Services LLC, AN2 Therapeutics, Inc., Andira Pharmaceuticals, Astellas Pharma, Inc., AstraZeneca, Basilea Pharmaceutica AG, Beckman Counter, Inc., bioMérieux, BioVersys AG, Blacksmith Medicines, Center for Discovery and Innovation, Cepheid, Chemia Biosciences Inc., Crestone, Inc., CytoSpar, LLC, Elion Therapeutics, Ellison Institute, Fedora Pharmaceuticals, FlightPath Biosciences, GARDP Foundation, Genentech, GenSci, GlaxoSmithKline plc, Gradientech, Hartford Hospital, Harvard University, ICON Laboratory Services, Inc., Innoviva Specialty Therapeutics, Inc., Institute for Clinical Pharmacodynamics, Iterum Therapeutics plc, Kaizen Biosciences, Kissaki Biosciences, LabConnect, LLC, Locus Biosciences, Inc., Meitheal Pharmaceuticals, Inc., Macro Biologics, Melinta Therapeutics, Merck & Co., MicrobiotiX, MicuRx Pharmaceutical Inc., Mundipharma International Ltd, National Institutes of Health, NovoBiotic Pharmaceuticals, LLC, Omnix Medical Ltd, Paratek Pharmaceuticals, Pattern Bioscience, Inc., Pfizer, Inc., Polaroid Therapeutics AG, PolyPid Ltd, PPD Global Central Labs, LLC, Pulmocide Ltd, Qiagen Sciences LLC, Qpex Biopharma, Inc., Revagenix, Inc., Roche Holding AG, Sandoz AG, Scynexis, Inc., SeLux Diagnostics, Shionogi & Co., Ltd, Specific Diagnostics, Spero Therapeutics, Thermo Fisher Scientific, US Food and Drug Administration, UT Southwestern Medical Center, Wockhardt Bio AG and Zoetis, Inc. D.D. is an employee of AbbVie and holds stocks of AbbVie.

## Supplementary data

Figure S1 is available as Supplementary data at *JAC-AMR* Online.

## References

[1] Kadri SS, Adjemian J, Lai YL et al Difficult-to-treat resistance in Gram-negative bacteremia at 173 US hospitals: retrospective cohort analysis of prevalence, predictors, and outcome of resistance to all first-line agents. Clin Infect Dis 2018; 67: 1803–14. 10.1093/cid/ciy378 PMC6260171 30052813

[2] Walker MK, Yek C, Sarzynski S et al Survival trends in patients with difficult-to-treat, antibiotic-resistant, Gram-negative infections in the era of next-generation antibiotics in the USA: a retrospective cohort study. Lancet Infect Dis 2026; 26: 659–70. 10.1016/S1473-3099(26)00020-4 PMC13268009 41903557

[3] Ozdede M, Balli Turhan FN, Geridonmez O et al Comparative nephrotoxicity of polymyxin B versus colistin: evidence from ensemble learning and propensity score matching. J Antimicrob Chemother 2026; 81: dkag044. 10.1093/jac/dkag044 42115839

[4] Prasad P, Sun J, Danner RL et al Excess deaths associated with tigecycline after approval based on noninferiority trials. Clin Infect Dis 2012; 54: 1699–709. 10.1093/cid/cis270 PMC3404716 22467668

[5] Tamma PD, Heil EL, Justo JA et al Infectious Diseases Society of America 2024 guidance on the treatment of antimicrobial-resistant gram-negative infections. Clin Infect Dis 2024: ciae403. 10.1093/cid/ciae403 39108079

[6] Sader HS, Mendes RE, Carvalhaes CG et al Changing epidemiology of carbapenemases among carbapenem-resistant Enterobacterales from United States hospitals and the activity of aztreonam-avibactam against contemporary Enterobacterales (2019-2021). Open Forum Infect Dis 2023; 10: ofad046. 10.1093/ofid/ofad046 PMC9945928 36846612

[7] Sader HS, Kimbrough JH, Doyle TB et al Frequency, antimicrobial susceptibility, and molecular characterization of Carbapenem-resistant Enterobacterales stratified by United States census divisions: results from the INFORM program (2018-2022). Open Forum Infect Dis 2025; 12: ofaf005. 10.1093/ofid/ofaf005 PMC11986335 40225828

[8] Tsai YV, Bookstaver PB, Kohn J et al The prevalence of gram-negative bacteria with difficult-to-treat resistance and utilization of novel beta-lactam antibiotics in the southeastern United States. Antimicrob Steward Healthc Epidemiol 2024; 4: e35. 10.1017/ash.2024.26 PMC10945936 38500714

[9] Carmeli Y, Cisneros JM, Paul M et al Aztreonam-avibactam versus meropenem for the treatment of serious infections caused by Gram-negative bacteria (REVISIT): a descriptive, multinational, open-label, phase 3, randomised trial. Lancet Infect Dis 2025; 25: 218–30. 10.1016/S1473-3099(24)00499-7 39389071

[10] Daikos GL, Cisneros JM, Carmeli Y et al Aztreonam-avibactam for the treatment of serious infections caused by metallo-beta-lactamase-producing Gram-negative pathogens: a phase 3 randomized trial (ASSEMBLE). JAC Antimicrob Resist 2025; 7: dlaf131. 10.1093/jacamr/dlaf131 PMC12301880 40727714

[11] CLSI . M07Ed12. Methods for Dilution Antimicrobial Susceptibility Tests for Bacteria that Grow Aerobically. Clinical Laboratory Standards Institute, 2024.

[12] CLSI . M100Ed36. Performance Standards for Antimicrobial Susceptibility Testing; 36th Informational Supplement. Clinical Laboratory Standards Institute, 2026.

[13] Castanheira M, Kimbrough JH, DeVries S et al Trends of beta-lactamase occurrence among *Escherichia coli* and *Klebsiella pneumoniae* in United States hospitals during a 5-year period and activity of antimicrobial agents against isolates stratified by beta-lactamase type. Open Forum Infect Dis 2023; 10: ofad038. 10.1093/ofid/ofad038 PMC9907474 36776778

[14] EUCAST . Breakpoint tables for interpretation of MICs and zone diameters v. 16.0. European Committee on Antimicrobial Susceptibility Testing. 2026. Available at: www.eucast.org/bacteria/clinical-breakpoints-and-interpretation/clinical-breakpoint-tables/. (Accessed March 13, 2026).

