# Supplementary material for: Activity of aztreonam–avibactam and comparators against difficult-to-treat resistant Enterobacterales from United States medical centres (2020–24)
Source: JAC Antimicrob Resist. 2026 Nov 5;8(6):dlag249. doi: 10.1093/jacamr/dlag249 (PMC13736119; doi:10.1093/jacamr/dlag249)
Supplement: Supplementary file 1 [file dlag249_supplementary_data.docx]

**Figure S1**. Susceptibility stratified by β-lactamase type

% Susceptible

| Carbapenemase (no. of isolates) | ATM-AVI | CAZ-AVI | MEM-VAB | IMI-REL | Cefiderocol |
| --- | --- | --- | --- | --- | --- |
| KPC (n=223)a | 100 | 99.6 | 99.6 | 98.2 | 99.1 |
| MBL (n=113) | 98.2 | 5.3 | 8.8 | 1.8 | 84.1 |
| Other CBases (n=22)b | 95.5 | 95.5 | 36.4 | 4.5 | 95.5 |
| No CBase (n=92) | 89.1 | 93.5 | 85.9 | 83.7 | 93.5 |

^a^ Exclude MBL co-producers.

^b^ Includes OXA-48-like (14), SME type (7), and IMI-4 (1) and excludes MBL co-producers.

Abbreviations: KPC, *Klebsiella pneumoniae* carbapenemase; MBL, metallo-β-lactamases; CBase, carbapenemase; ATM-AVI, aztreonam-avibactam; CAZ-AVI, ceftazidime-avibactam; MEM-VAB, meropenem-vaborbactam; IMI-REL, imipenem-relebactam.
